# Combined Supra- and Sub-Lesional Epidural Electrical Stimulation for Restoration of the Motor Functions after Spinal Cord Injury in Mini Pigs

**DOI:** 10.3390/brainsci10100744

**Published:** 2020-10-16

**Authors:** Filip Fadeev, Anton Eremeev, Farid Bashirov, Roman Shevchenko, Andrei Izmailov, Vage Markosyan, Mikhail Sokolov, Julia Kalistratova, Anastasiia Khalitova, Ravil Garifulin, Rustem Islamov, Igor Lavrov

**Affiliations:** 1 Department of Medical Biology and Genetics, Kazan State Medical University, 420012 Kazan, Russia; philip.fadeyev@gmail.com (F.F.); faridbashirov@yandex.ru (F.B.); shev42006@yandex.ru (R.S.); gostev.andrei@gmail.com (A.I.); vage.markosyan@gmail.com (V.M.); supermihon@yandex.ru (M.S.); Juliya2199@yandex.ru (J.K.); knt-15.04@yandex.ru (A.K.); ravil.l16rus@mail.ru (R.G.); 2 Institute of Fundamental Medicine and Biology, Kazan (Volga Region) Federal University, 420008 Kazan, Russia; 2anton.eremeev@mail.ru; 3 Department of Neurology, Mayo Clinic, Rochester, MN 55905, USA; 4 Department of Biomedical Engineering, Mayo Clinic, Rochester, MN 55905, USA

**Keywords:** spinal cord injury, mini pigs, epidural electrical stimulation, translesional network

## Abstract

This study evaluates the effect of combined epidural electrical stimulation (EES) applied above (C5) and below (L2) the spinal cord injury (SCI) at T8–9 combined with motor training on the restoration of sensorimotor function in mini pigs. The motor evoked potentials (MEP) induced by EES applied at C5 and L2 levels were recorded in soleus muscles before and two weeks after SCI. EES treatment started two weeks after SCI and continued for 6 weeks led to improvement in multiple metrics, including behavioral, electrophysiological, and joint kinematics outcomes. In control animals after SCI a multiphasic M-response was observed during M/H-response testing, while animals received EES-enable training demonstrated the restoration of the M-response and H-reflex, although at a lower amplitude. The joint kinematic and assessment with Porcine Thoracic Injury Behavior scale (PTIBS) motor recovery scale demonstrated improvement in animals that received EES-enable training compared to animals with no treatment. The positive effect of two-level (cervical and lumbar) epidural electrical stimulation on functional restoration in mini pigs following spinal cord contusion injury in mini pigs could be related with facilitation of spinal circuitry at both levels and activation of multisegmental coordination. This approach can be taken as a basis for the future development of neuromodulation and neurorehabilitation therapy for patients with spinal cord injury.

## 1. Introduction

Spinal cord injury (SCI) remains one of the most difficult problems in biomedical science [1,2,3]. Currently, the quality of life and life expectancy of patients with SCI largely depend on symptomatic treatment [4,5,6]. Electrical stimulation of the spinal cord has been extensively used as a complementary approach for rehabilitation of patients with SCI [7,8,9,10,11,12,13,14]. The role of the specific neural targets and parameters of stimulation in the restoration of the motor functions has been described previously in animals and in humans [15,16,17,18,19]. To date, however, there is no consistent protocol for spinal cord stimulation after SCI available for clinical practice [16]. Over the last decade, epidural electrical stimulation (EES) has been successfully implemented as a method for enabling intrinsic activity of the spinal cord circuitry facilitated with sensory inputs [15,20,21,22,23,24]. There is also growing evidence of the positive effects of EES on neuronal plasticity following SCI [25,26,27]. Even in an isolated *in vitro* models or in freely moving *in vivo* models, EES or neuroactive substances applied at the lumbar segments can facilitate well-coordinated bilateral rhythmic activity [20,22,23,28,29,30]. In clinical studies, patients with motor complete spinal cord injury (American spinal cord association impairment scale AIS-A and B) in presence of EES were able to control volitional motion and independent walking, indicating discomplete type of injury [12,13]. EES applied at the different spinal segments can modulate local neural networks and related functions [16,31]. The propriospinal network provides coordination between multiple spinal segments and between upper and lower limbs during complex motor patterns like locomotion [25] and likely involved in the coordination of the sensorimotor information along with activation of the local spinal network for upper and lower extremities for synergistic coordination of motor outcome [15,16,32]. Multiple evidence suggests that spinal cord circuitry above and below the SCI could participate in the restoration of the sensorimotor function after SCI [33,34,35,36,37]. At the same time, the effect of spinal cord stimulation applied above or combined stimulation applied above and below SCI remains virtually unknown. In addition, the vast majority of animal experiments studied the effect of EES on the spinal neural network on small animal models, primary rodents, and given their anatomical and physiological differences with a human, it is expected that large animals may provide closer to human model of SCI, particularly in relation to the effect of EES [31].

The epidural electrical stimulation is nowadays intensively explored technique for functional restoration after spinal cord injury in animals and human. Animal studies conducted by our and other groups over last decade demonstrated positive effect of epidural stimulation on multifunctional restoration after severe SCI. At the moment, most of the results in this new and rapidly growing field, were focusing on facilitation of sub-lesional spinal circuitry to target central pattern generators responsible for lower extremities function. Multiple evidences presented in previous studies [12,38,39] demonstrate the role of the afferent fibers crossing the SCI and modifying the supra-lesional network, stimulating integration of sensorimotor information coming to the spinal network below, above the injury, and from supraspinal influence. In this study we evaluated potential of spinal cord stimulation to engage various components that comprise the trans-lesional network, their functional recovery after SCI, and the implications on development of future neuromodulation, rehabilitation, and neuroprosthetics technologies. Our recent study demonstrates that even after complete SCI, limited connectivity across newly growing fibers through the biomaterial scaffold could modulate sub-lesional circuitry and improve motor functions [39]. Together these findings inform a hypothesis that combined sub- and supra-lesional electrical stimulation accompanied with locomotor training may improve the functional restoration. In this study we evaluated the effect of combined EES applied above and below the contusion injury in mini pigs with the following EES-enabled motor training.

## 2. Materials and Methods

Experiments were performed on six 15–20 kg male Vietnamese swine. Animals were placed in individual cages under standard laboratory conditions with unlimited access to food and water and a 12 h day/night cycle. The protocol of the experiment, including anesthesia, surgery, postoperative care, testing, and euthanasia, was approved by the Animal Care Committee of Kazan State Medical University (#2.20.02.18 of 20 February 2018). All experimental procedures were performed in accordance with the standards to minimize animal suffering and the size of experimental groups. Animals were included in experiments after a period of adaptation of at least 7 days. Animals were divided into the following experimental groups: control (C-group)—swine after SCI (contusion spinal injury) received motor rehabilitation therapy on a treadmill (*n* = 2); treated (SCI-EES-group)—animals with SCI subjected to EES-enabled motor training on a treadmill (*n* = 2); and I-group—intact animals (*n* = 2) used to collect baseline outcome of behavioral and electrophysiological studies. The animals were sacrificed on 8th week after SCI. The euthanasia was performed under deep anesthesia by overdosing of inhalational narcosis agent (Isoflurane). 

### 2.1. Surgical Procedures

12 h before the surgery, water and food were removed from animals. 1 h before surgery, the animal was injected intramuscularly with the antibiotic Baytril 2.5% (Bayer, Vladimir, Russia) 0.2 mL/kg. Immediately before the surgery, Zoletil 100 (Virbac Sante Animale, Carros, France) was intramuscularly administered as induction anesthesia at a dose of 10 mg/kg. When the required sedation level was achieved, the animal was placed on the operating table and anesthesia was maintained using inhalation apparatus (Minor Vet Optima, Zoomed, Moscow, Russia) with isoflurane (Laboratorios Karizoo, S.A., Barcelona, Spain) as 2.0%–2.5% mixture with oxygen (oxygen alone was started at 3–5 min prior to procedure completion). Operating fields were cleaned from hair and treated with antiseptic solutions (10% solution of povidone-iodine) (Hemofarm, Vrsac, Serbia), then twice with an alcohol solution for external application chlorhexidine bigluconate 0.05% (LLC “Rosbio,” St. Petersburg, Russia). During the procedure, the body temperature of the animal was maintained at 38 °C.

### 2.2. Implantation of Stimulating and Reference Electrodes

A skin incision was performed on the neck for access to the cervical vertebrae and on lumbar region to reach lumbar vertebrae. The vertebrae C3 to C5 and Th14 to L3 were released layer-by-layer and laminectomies (C4–5 and L1–2) were performed to expose the dura mater.

The wires were conducted through prepared additional “windows” in yellow ligament between the C3–C4 and T14-L1 to lead the stimulating electrodes under intact vertebral laminas for further safe fixation to dura mater. Epidural electrodes at both sites were placed so that the distal end of the wire was under the arc of the underlying vertebra. The stimulating electrodes with circularly exposed insulation (AS362, Cooner Wire Company, Chatsworth, CA, USA) were fixed to the dura mater with Prolene 7/0 synthetic sutures at a distance of about 5 mm at the midline of the spinal cord under the control of surgical microscope.

The loops of wire were formed to prevent tension during movements and placed under superficial fascia.

The reference electrodes were implanted intramuscularly into the neck muscles and in m. erector spinae at the lumbar region. The wires were connected to a 12-channel connector (Omnetics Connector Corporation, Minneapolis, MN, 55432-USA) for connection to electrophysiological equipment. The connector was placed on the dorsal surface of the animal body in the area of the skin and fixed to the skin with ligatures. All incisions were sutured layer by layer. Postoperative care included the necessary (antibacterial, analgesic, infusion) therapy: Cephazolin (RUP “Belmedpreparations” Minsk, Belarus) 1 gr/5 mL, QD, IM, 7 days (dilute single dose vial with 3 mL of sterile water for injection and 2 mL of 2% lidocaine); Ketorol (“Dr. Reddy ‘s, “, Hyderabad, India) 30 mg/mL, BID, IM, 7 days; NaCl 0.9% Solution 400.0 mL QD, SC, 5 days. The dressing on the postoperative wound was changed daily. The recovery period lasts for at least 7–10 days.

### 2.3. Contusion Injury

At 7–10 days after electrodes implantation, contusion injury of the spinal cord was performed as described by Lee et al. [40]. The reason for using the ‘free fall’ contusion model is related to reproducibility and simple performance, as well as with a large amount of previously published results using this approach. The animal has undergone sequentially all stages of pre-operative preparation as described previously (section: Surgical procedures). Prior to spinal cord injury, an 18 Fr silicone urinary catheter was inserted in order to retract urine during surgery and early postoperative period. At the surgery, about 400.0 mL of Ringer’s solution was infused through a venous catheter inserted into the ear vein. The venous catheter was left in the vein for subsequent postoperative infusions. Contusion was performed as follows: the animal was fixed with the straight spine extended. A longitudinal section at T7–T12 vertebras was performed and processes and arcs of the vertebrae T7–T9 were released from soft tissues. Then, interosteal and perosteal ligaments were dissected. Laminectomy of T8 and T9 vertebras was performed next to expose the spinal cord. A metal impactor cylinder was placed in and contusion injury was performed with a 50 g weight falling from a height of 50 cm.

### 2.4. Postoperative Care

All animals received proper antibacterial, analgesic, infusion, and vitamin therapy with thiamine (B1)/Pyridoxin (B6) (JSC “Borisovsky Medical Drug Factory” of Borisovsk, Belarus,)—2 mL, QD, SC, 10 days; Cyanocobalamine (B12) (OAO “Yerevan Chemical and Pharmaceutical Company” Yerevan, Armenia)—2 mL, QD, IM, 10 days; Ascorbic acid (FKP “Armavir bio-factory” Armavir, Russian Federation)—4 mL, QD, IM, 10 days; Riboxin (OAO “Dalchimfarm”, Khabarovsk, Russian Federation)—5 mL, QD, IM, 10 days; Klaforan (cefotaxim) (“Krasnfarma” Krasnoyarsk, Russian Federation)—1 gr/5 mL, 1 QD, IM, 10 days; Ringer’s solution—400.0 mL, QD, SC, 3–5 days; Aktovegin (Takeda, Yaroslavl, Russian Federation)—2 mL, 1 QD, IM, 3 days; Dimedrol (Dalchimfarm JSC, Khabarovsk, Russian Federation)—1 mL, QD, IM 10 days; Prozerin (OAO “Nowibchimfarm” of Novosibirsk, Russian Federation)—0.5 mL, QD, SC, 10 days; Dexamethasone (“KRKA” of Novo-mesto, Slovenia)—1 mL, QD, SC 10 days. Postoperative wound dressing and control of the implant condition was performed daily. In postoperative period monitoring of experimental animal condition included assessment of postoperative wound condition, presence of urine passage along the urinary catheter, the appearance of defecation, appetite, expression of indicative reflex, presence or absence of motor activity.

### 2.5. Training on the Treadmill

At 2 weeks before the surgery, and from 2 to 8 weeks after SCI, animals underwent motor training on a treadmill. The training was performed every second day for 30 min in the morning and in the evening with 7–8 h difference. During training, animal was fixed with a bandage around the torso. The load on the hind limbs was maintained at 5% to 20% of animal weight. The speed of the treadmill belt was 0.3–0.4 m/s. If the animals did not show independent locomotor activity with their hind limbs, the researcher moved the hind limbs of the animals, simulating walking. Motor training in swine from the SCI-EES group was combined with EES applied at C5 and L2.

### 2.6. Epidural Stimulation

EES was performed in animals from the SCI-EES group during treadmill training from 2 to 8 weeks after SCI both at C5 and L2 levels. Stimulation at C5 was aimed to facilitate the activity of neurons above the injury and provide required interaction between neural networks related to the forelimbs above and spinal network below the injury. Stimulation at L2 level was applied to activate central pattern generators to stimulate active hind limbs movement. For EES a Digitmer DS7A (Digitmer Ltd., Welwyn Garden, UK) was used. Parameters of EES were selected, so animals produced walking movements matched with the speed of the treadmill belt (Supplementary Video 1). The current used for EES was adjusted to not cause any discomfort and was selected individually for each animal. Stimulation was considered successful when tonic muscle response was observed below the injury and facilitated the walking movements of the hind limbs on a treadmill. During the morning session, stimulation was performed for half an hour above the injury site at the C5 vertebra level, with the current intensity selected individually for each animal based on our previous findings and behavioral signs, indicating absence of discomfort, which usually was in range 7–15 mA, frequency 20–25 Hz, pulse duration 0.2 ms. During the evening session, for half an hour, spinal cord stimulation was performed below the injury site at the L2 vertebra level on optimal motor performance with EES in body-weight support. The current, in this case, was 13–25 mA, frequency 20–25 Hz, and pulse duration of 0.2 ms.

### 2.7. Electrophysiological Assessment

In this study, the motor evoked potentials (MEP) induced by EES applied at C5 and L2 levels were recorded in soleus muscles before and two weeks after SCI to assess potential effect of stimulating electrodes. At the end of the study, at 8 weeks, we evaluated M-response and H-reflex by stimulating sciatic nerve to assess changes in segmental excitability. Before the electrophysiological assessment, the animals were anesthetized and connected to an inhalation apparatus. Digitimer DS7A (Digitimer Ltd., Welwyn Garden, UK), amplifier with filters ranging from 5 Hz to 2 kHz (Biosignal amplifier, g.tec medical engineering GmbH, Schieldberg, Austria), and LabChart data collection and analysis systems (AD Instruments Inc., Colorado Springs, CO, USA) were used in this study. Before and 2 weeks after the spinal cord injury, electrophysiological testing was carried out. Motor evoked potentials were recorded from the soleus muscle of both hind limbs during epidural stimulation at the C5 and L2 vertebra level. Stimulation was performed by single rectangular pulses using pre-implanted electrodes (described previously). The intensity of stimuli was 8–92 mA, duration was 0.2 ms and frequency was 1 pulse in 30 s. Muscle responses were recorded using needle electrodes (stainless steel, diameter was 0.6 mm, and length was 50 mm), which were injected into the soleus muscle at the point of divergence of medial and lateral peritoneal muscles. Prior to spinal cord injury and 8 weeks after the injury, H-reflex and M-response were recorded in the soleus muscles of both hind limbs caused by sciatic nerve stimulation. For this purpose, stimulating electrodes (stainless steel, diameter was 0.6 mm, and length was 50 mm) were introduced into the area of the sciatic nerve projection (2 cm below the large trochanter of the femur 1 cm down from the femur). Stimulation was performed with a single rectangular pulse. The intensity of stimuli was 3–64 mA, duration was 0.2 ms, and frequency was 1 pulse in 30 s. During each testing, the position of the electrodes, as well as the angles in the joints of the hind limbs, were controlled to exclude the influence of these parameters on the recorded muscle responses. At each stimulation intensity, at least 5 pulses of current were applied, and the responses averaged. Threshold, latency, maximum amplitude, and duration of evoked potentials were evaluated. The evoked potentials were evaluated when the response amplitude was maximum for M response and H reflex (with an average stimulation intensity of 64 ± 12 mA).

### 2.8. Joint Kinematics

Video analysis of joint kinematics was evaluated based on changes in angles in hip, knee, and ankle joints while animals were walking on a treadmill (2 weeks before and weekly after spinal cord injury, from 5 to 8 weeks). Colored marks were applied in projections of the ridge of the iliac bone, trochanter of the femur, knee, ankle joint, and hoof. Video recording during walking was performed with Canon PowerShot S5 IS camera (Canon, Tokyo, Japan). Each recording session lasted for 10–15 s, with 5 walking cycles recorded. The recording was performed by placing the animal on a treadmill in the absence of epidural electrical stimulation, as well as in motor training combined with spinal cord stimulation. The video records were used to analyze the hip, knee, and ankle joints movement range. Video analysis of joint kinematics was performed using Kinovea software 0.8.25. In this program, the joint movement range was calculated as a difference between the maximum and minimum joint angles when evaluating 5 walking cycles. R 3.4.4 (R Foundation for Statistical Computing, Vienna, Austria) was used to analyze and visualize the results of the joint kinematics study.

### 2.9. Behavioral Test

At 2 weeks before and weekly after spinal cord injury, recovery of motor function was assessed with the scale of pig behavior Porcine Thoracic Injury Behavior Scale (PTIBS), according to which the motor function of the hind limbs was characterized ranging from no hind limb movements (score 1) to normal walking (score 10). PTIBS scores 1–3 reflected “hind limb drag”, score 4–6 reflected varying degrees of ability to “step,” and score 7–10 reflected varying degrees of ability to “walk” [38].

### 2.10. Data Analysis

We did not perform hypotheses testing and estimation procedures using collected data due to limited sample size consideration. Average values presented in the article are intended for descriptive purposes only.

## 3. Results

### 3.1. Motor Potentials Evoked by EES

**Figure 1:**
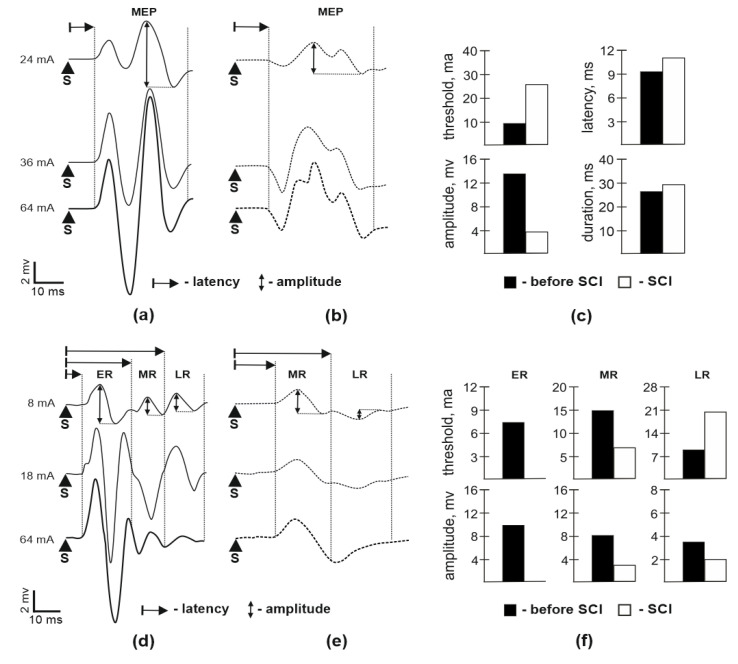
Motor evoked response potentials (MEP) in soleus muscle during spinal cord stimulation. (**a**)—MEP in soleus muscle during stimulation at the cervical segment C5 before spinal cord injury (SCI); (**b**)—MEP in soleus muscle during stimulation at C5 segment 14 days after SCI; (**c**)—comparison between the different parameters of MEP in soleus muscle during stimulation at cervical segment C5; (**d**)—responses in soleus muscle during stimulation at the lumbar segment L2 before SCI; (**e**)—responses in soleus muscle during stimulation at the lumbar segment L2 14 days after SCI; (**f**)—comparison between the different parameters of early response (ER), middle response (MR), and late response (LR) in soleus muscle during stimulation of at the lumbar segment L2. S—stimulus. Data obtained for each animal are presented as points, bars represent group-wise average values.

The motor evoked potentials (MEP) recorded during EES (intensity 8–90 mA, duration 0.2 ms, frequency 1 pulse in 30 s) at C5 and L2 levels were recorded in soleus muscles before contusion injury and 14 days after SCI before starting EES treatment (Figure 1). During stimulation at C5 before SCI, the amplitude of MEP gradually increased with stimulation intensity increasing, reaching a maximum (Figure 1a). At 14 days after SCI, stimulation at C5 caused multicomponent MEP with similar dynamics, but with reduced maximum amplitude (Figure 1b,c). During stimulation at L2 prior to SCI, early, middle, and late responses were recorded (Figure 1d). The amplitude of the early response increased as the intensity of stimulation increased, reaching a maximum, and did not change with further increase in stimulation intensity, the amplitude of the middle and late responses increased, reaching a maximum, and then decreased with increase in stimulation intensity. At 14 days after SCI, stimulation at L2 did not evoke early response (Figure 1e) and the amplitude of the middle and late responses was decreased (Figure 1f).

### 3.2. M-Response and H-Reflex Evaluation

**Figure 2:**
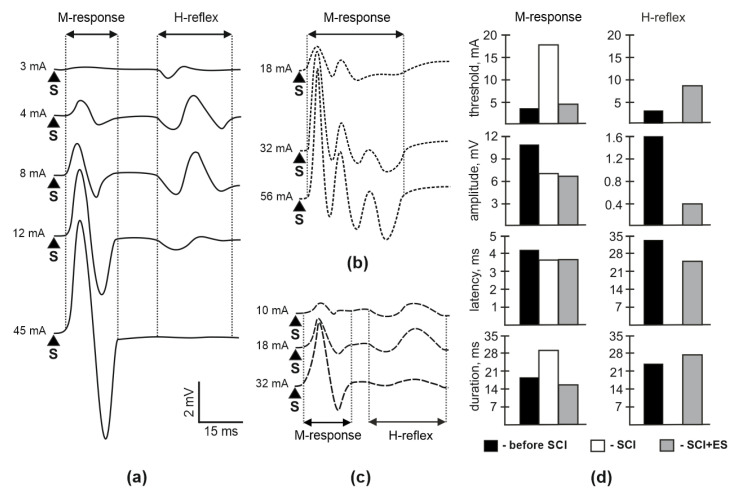
The responses in soleus muscle induced by sciatic nerve stimulation. (**a**)—the response of soleus muscle before SCI; (**b**)—response of soleus muscle 8 weeks after SCI in SCI group; (**c**)—response of soleus muscle 8 weeks after SCI in SCI-ES (electrical stimulation) group; (**d**)—comparison between the different parameters of M-response and H-reflex. S—stimulus. Responses in soleus muscle recorded before SCI (before SCI), after SCI (SCI), and in SCI + EES (epidural electrosimulation) group (SCI-EES).

Assessment prior to the spinal cord injury demonstrated a short-latency direct motor M-response and a monosynaptic H-reflex in soleus muscle. The shape and latency of recorded potentials changed during a gradual increase in stimulus intensity (Figure 2a–c). At 8 weeks after SCI a multiphasic M-response was observed (Figure 2b) with increased threshold to 450%, lower amplitude to 68%, and with an increased duration to 174% (Figure 2d). In animals from SCI-EES-group, both M-response and H-reflex were recorded (Figure 2c). The restoration of the standard shape of the M-response may reflect the synchronous involvement of the motor units. However, the amplitude of the response was different compared to the results collected before SCI. The amplitude of M-response was decreased to 60% the threshold of H-reflex increased to 229% the amplitude of H-reflex decreased to 25%, and latency to 74%, while the duration of H-reflex increase to 120% (Figure 2d).

### 3.3. Video Analysis of Hind Limb Joints Kinematics

**Figure 3:**
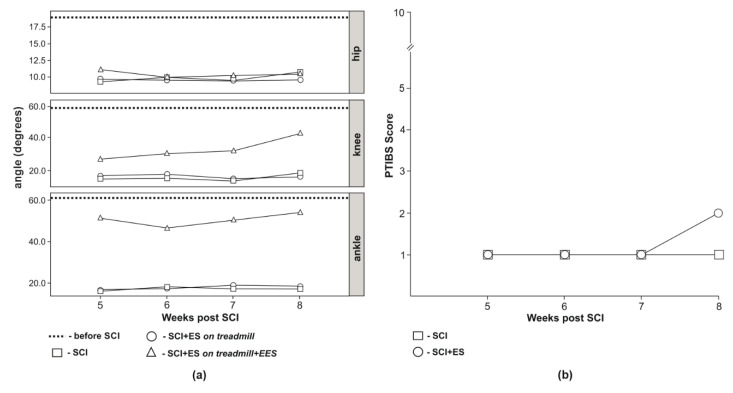
Analysis of the movements in the mini pig hind limbs. (**a**)—kinematics analysis of the movements in the joins of the hind limbs (average of 5 steps): the range of motion in hind limbs before SCI (before SCI), after SCI (SCI), and in group with SCI + EES on a treadmill (SCI-EES). SCI + EES on treadmill + EES, the range of movement in hind limb joints during a motor performance with EES in SCI-EES. (**b**) motor activity in mini pig after spinal cord injury assessed with PTIBS scale: SCI–PTIBS (Porcine Thoracic Injury Behavioral Scale) assessment in SCI group; SCI + EES−PTIBS assessment in SCI−EES group.

Prior to SCI hind limb kinematics analysis demonstrated hip joint movement in the range of 20.25 degrees, knee joint—59.00 degrees, and ankle joint—63.25 degrees (Figure 3a). Posterior extremities kinematics analysis of animals from the SCI group demonstrated a decrease of the range compared to intact animals (I-group). Thus, the hip joint movement range 5 weeks after SCI was 9.00, 6 weeks—10.00, 7 weeks—9.50, and 8 weeks—10.50 degrees; the knee joint movement range—11.00, 10.50, 10.00, and 18.50 degrees after 5, 6, 7, and 8 weeks post-injury, respectively. Ankle joint movement range 5 weeks after SCI was 7.50 after 6 weeks—9.00, after 7 weeks—7.50, and 7.50 degrees 8 weeks after SCI (Figure 3a). Animals from the SCI-EES-group placed on a treadmill without epidural stimulation had the same pattern of the joint kinematics as the animals from SCI-group that was significantly reduced compared to animals in I-group (Figure 3a). The hip joint movement range 5 weeks after SCI was 9.33, 6 weeks—9.00, 7 weeks—9.33, and 8 weeks—9.33 degrees; knee joint movement range was 13.33, 15.33, 10.00, and 12.67 degrees after 5, 6, 7, and 8 weeks post-injury, respectively; ankle joint movement range was 6.67, 8.33, 11.00, and 10.00 degrees, correspondingly to 5, 6, 7, and 8 weeks after SCI (Figure 3a). Animals from SCI-EES group demonstrated hind limb locomotor activity only with L2 stimulation. With EES at L2 animals were able to lift the pelvis and perform walking movements throughout 30 min of EES session. In animals from SCI-EES-group during treadmill training accompanied with epidural stimulation at L2 at 5 weeks after SCI the hip joint movement range was 11.33, 6 weeks—9.33, 7 weeks—10.00, and 8 weeks—10.33 degrees and did not differ from SCI-group. The knee joint movement range was significantly increased compared to SCI-group and was 27.33, 30.00, 31.33, and 44.33 degrees, correspondingly at 5, 6, 7, 8 weeks after SCI. The ankle joint movement range was also significantly increased in comparison to SCI-group and was 55.33, 48.00, 54.00, and 58.33 degrees correspondingly at 5, 6, 7, 8 weeks post-injury (Figure 3a). The comparison of SCI- and SCI-EES-groups suggests that a combination of physical training with epidural stimulation leads to a statistically reliable increase of knee and ankle joints movement range at all phases of this study.

### 3.4. Behavioral Assessment

Assessment with PTIBS motor recovery scale demonstrated that in animals from the SCI group, the motor performance was consistent with 1 point (no active hind limb movements, the sacrum, and knees on the ground) and did not change during 8 weeks after SCI. In animals from SCI-EES-group 8 weeks after injury active hind limb movements were observed and motor function was rated at two points (Figure 3b).

## 4. Discussion

Currently available therapeutic options unable to provide significant improvement in the quality of life of SCI patients. Most of the studies of SCI were conducted on small laboratory animals (mice and rats) that have similarities in the organization of the motor system and the spinal network generating rhythmic movements (central pattern generator, CPG) found in most of the vertebrates, including humans [7,39,40,41]. At the same time, rodents compare to large mammals have a smaller brain and spinal cord sizes and shorter conductive pathways of neural fibers [38]. Several other metrics collected on rodents were found to be different from humans [42,43,44]. For example, activation of sensory feedback in rats may significantly improve the motor function after severe SCI, which is usually not a case in humans with severe SCI [44]. Translational large animal models can facilitate the development and testing of new therapeutic techniques where the difference between rodents and humans should be considered [45].

Currently, motor training and spinal cord stimulation are the most effective therapeutic techniques for recovery of sensorimotor function after SCI. Multiple studies report that the combination of rehabilitation strategies may increase functional recovery after SCI [16,41]. A key component of rehabilitation is motor training that activates afferent signaling to the spinal cord, critical for generating an adequate motor output [46,47,48,49]. EES has been demonstrated as an effective technique in facilitating walking patterns in cats [15,28,50,51] and rats [22,52,53,54], as well as in humans after functionally complete SCI [7,9,55]. The effect of EES is likely related to the interneuronal spinal network that integrates and efficiently processes the range of somatosensory inputs required to generate the appropriate motor task [15,21,22,32,56] and also was linked with activation of local spinal neuronal groups [57] or with real-time assessment of the local blood flow [58,59]. The upper lumbar segments may play a key role in facilitation of the motor tasks, at the same time, interacting with the multi-segmental networks to inform unique motor outcome [32]. The restoration of the motor function after SCI largely depends on supraspinal control [60] and the functional connectivity coordinating the extremities in quadripedal animals [39,61,62,63,64], as well as between hands and legs in humans [65,66,67,68], attributed to the propriospinal system. The propriospinal network of the spinal cord is responsible for interaction between multisegmental and particularly between cervical and lumbar locomotor networks [69] and capable of activation of spinal interneurons involved in complex motor control [70,71]. In non-traumatic human volunteers, transdermal stimulation of the spinal cord at lumbar segments facilitated rhythmic activity in the lower limbs. A combination of stimulation of the cervical segments with stimulation of the lumbar segments resulted in improvement in kinematics, while cessation of cervical stimulation resulted in a progressive decline of the walking pattern [32].

The recovery of motor functions after complete SCI was previously attributed to the influence of the downstream pathways indirectly conducting supraspinal signaling through the site of the injury [38,72,73]. With a clinical diagnosis of complete SCI, residual subfunctional connections are capable of transmitting supraspinal influence below the injury level. This profile of trauma is known as “incomplete” [72,73]. The role of this residual subfunctional connectivity after SCI and mechanisms of its reactivation by electrical stimulation remains largely unknown [16,74]. The neural networks below and above the injury may form a translesional network, which can reorganize over time in functional neuronal structure involved in the restoration of the motor functions, such as volitional motor control with EES. It is expected that electrical stimulation of the spinal networks above the injury may facilitate the activity of the neuronal circuitry below the injury, particularly when downstream pathways from the brain are unable to effectively grow through the injury and provide required interaction between neural networks above and below the injury [33,34,35,36,37]. The propriospinal system consists of short and long axons that interconnect most of the spinal segments promoting modulation during the execution of the most motor commands [75,76], which appears to be an important component in restoring functions in the absence of sufficient axons directly interacting with the corticospinal tracts. 

In this study, we implemented a new protocol with electrical epidural stimulation applied above, at C5, and below, at L2, the injury, combined with EES-enabled motor training for restoring the motor function after SCI in mini pigs. The stimulation at C5 in this model is expected to facilitate cervical network and promote the formation of new functional connections across supra-lesional and sub-lesional networks, compensating the luck of supraspinal control, and thereby, increasing the therapeutic effect of lumbar-sacral stimulation at L2. Selective increase and decrease in the activity of spinal neurons can respectively enhance or weaken spontaneous regeneration [77,78]. Some level of supraspinal influence across the injury on sub-lesional network was detected by recording of motor evoked potentials, caused by spinal cord stimulation above the injury site, at C5 segment. Observed changes in motor potentials two weeks after SCI may indicate on modulation in the functional state of translesional spinal network. The multicomponent response recorded during C5 stimulation could be related to the different conduction through the efferent fibers in the spinal cord and/or the different speed of recruitment of the motor units. The future studies are required to determine the nature of responses recorded with stimulation at C5. The nature of the evoked responses caused by epidural stimulation at L2 has been discussed in a number of previous studies [21,22,53]. The early response (ER) is likely related to direct activation of the efferent fibers or motor neurons, the middle response (MR) corresponds to monosynaptic potential, and late response (LR) likely involves the activation of a polysynaptic neural network, including at least three synapses [22]. Recorded 2 weeks after injury absence of ER and decrease in the amplitude of MR and LR may reflect a decrease in the excitability of the spinal locomotor network as a result of developing post-traumatic spinal shock. At 8 weeks after SCI we found changes in responses recorded during stimulation of the sciatic nerve in animals from experimental groups compared to results collected before the SCI. In animals with SCI, the M-response had a multicomponent shape and was recorded at the threshold intensities and up to the maximum stimulation intensity. The changes in M-response can reflect the post-traumatic reorganization of the motor units. It is known that functional unloading initiates muscles atrophic changes and particularly changes in the composition of the muscle [79] that can lead to a decrease in the number of motor units and their asynchronous recruitment during activation. The absence of H-response after SCI is likely associated with prolonged M response; thus, the late part of M-response may superimpose the H-response. In animals from the EES-trained group, the M-response curve with one negative and one positive peak reflects the synchronous involvement of the motor units. Increase the threshold and decrease the amplitude of H-response in treated animals may also indicate on decrease in the excitability of corresponding motoneurons. Previous electrophysiological studies demonstrated that spinal cord stimulation may alter the properties of motoneurons, reducing their excitability [80,81]. In addition, the stimulation of the sciatic nerve may activate most of the afferents, including skin afferents and afferents of antagonist muscles [82], which may have inhibitory influence on recorded activity, and activation of intraspinal inhibitory mechanisms following SCI contributes to the restoration of locomotor activity [83].

The results of the electrophysiological assessment in this study are supported by kinematic and behavioral tests. The volume of movement in the hip and knee joints in the SCI-EES group was greater, and in the ankle joint even approached the values recorded before the SCI. The relatively high amplitude of movements recorded in the ankle joint can be related to the restoration of the activity in anterior tibial muscle responsible for foot flexion. In mechanical unloading conditions, the most critical changes were primarily found in extensors, [84,85,86], in addition, movements in each joint can be coordinated by a separate neural network [87,88]. In this regard, it can be expected that the location of the stimulating electrode at L2 may facilitate a higher number of the motor neurons related to anterior tibial muscles, ensuring the restoration of flexion. Recovery of motor function assessed with PTIBS scale in the SCI-EES-group revealed active hind limb movements, while in the SCI group no active hind limb movements were found. The reliability of PTIBS assessment is considered high [38] and, in general, the PTIBS data in this study are in line with the results of EMG and hind limb joint kinematics analysis and demonstrate the improvement in motor function in mini pigs from the SCI-EES group. Thus, the therapeutic effects observed in this study with two-level (above and below the injury) electrical stimulation combined with EES-enabled motor training are likely to determine the functional gain.

## 5. Conclusions

In this study, behavioral test, analysis of joint kinematics, and electrophysiological evaluation, altogether demonstrate the positive effect of two-level (cervical, C5, above and lumbar, L2, below the injury) epidural electrical stimulation on functional restoration of the spinal cord in mini pigs following spinal cord severe contusion injury. These results can potentially be taken as a basis for the future development of a therapeutic protocol for neuromodulation and neurorehabilitation of patients with spinal cord injury.

## Supplementary Materials

The following are available online at https://www.mdpi.com/2076-3425/10/10/744/s1, Supplementary Video 1. Two representative mini pigs with spinal contusion during training on a treadmill without and with EES applied at L2 lumbar segment. Both animals demonstrate significant increase in motor control over hind limbs in presence of L2 electrical epidural stimulation used for EES-enabled training in this study.
